# Magnetic imaging of subseafloor hydrothermal fluid circulation pathways

**DOI:** 10.1126/sciadv.abc6844

**Published:** 2020-10-30

**Authors:** Christopher G. Galley, John W. Jamieson, Peter G. Lelièvre, Colin G. Farquharson, John M. Parianos

**Affiliations:** 1 Department of Earth Sciences, Memorial University of Newfoundland, St. John’s, NL A1B 3X7, Canada.; 2 Department of Mathematics and Computer Science, Mount Allison University, Sackville, NB E4L 1E2, Canada.; 3 Nautilus Minerals Inc., Brisbane, 4169, Australia.

## Abstract

Hydrothermal fluid circulation beneath the seafloor is an important process for chemical and heat transfer between the solid Earth and overlying oceans. Discharge of hydrothermal fluids at the seafloor supports unique biological communities and can produce potentially valuable mineral deposits. Our understanding of the scale and geometry of subseafloor hydrothermal circulation has been limited to numerical simulations and their manifestations on the seafloor. Here, we use magnetic inverse modeling to generate the first three-dimensional empirical model of a hydrothermal convection system. High-temperature fluid-rock reactions associated with fluid circulation destroy magnetic minerals in the Earth’s crust, thus allowing magnetic models to trace the fluid’s pathways through the seafloor. We present an application of this modeling at a hydrothermally active region of the East Manus Basin.

## Teaser

Magnetic studies of the seafloor have produced the first empirical model of a hydrothermal upflow zone.

## INTRODUCTION

Since their discovery on the East Pacific Rise in 1979 (*1*), high-temperature hydrothermal vents, and associated seafloor massive sulfide (SMS) deposits, have been identified as unique oases of chemosynthetic-based life-forms and provide key information to the understanding of hydrosphere/lithosphere heat transfer at mid-ocean ridges and ore-forming processes on the seafloor (*2*). Hydrothermal vents form as the end product of hydrothermal fluid convection systems driven by shallow magmatic heat sources (*3*). Subseafloor fluid convection draws seawater into the crust, where it is progressively heated and reacts with the host rock mineral assemblages before returning to the seafloor as a hot, reduced, metal- and sulfur-rich hydrothermal fluid. At the seafloor, the fluids mix with cold seawater, causing the precipitation of a portion (*4*) of the dissolved components at focused venting sites along faults and/or fractures (*5*). Over time, the resultant accumulation of metal-rich sulfide minerals at vent sites can result in the formation of SMS deposits and continually provide sustenance for the organisms that inhabit vent sites. Much of our understanding of hydrothermal systems is based on the study of known hydrothermal vents and associated deposits. However, the exposed vent sites represent only a small component of the overall hydrothermal system. To develop a holistic understanding of the size and subseafloor geometry of hydrothermal systems, previous studies have relied on numerical modeling (*6*, *7*), seafloor heat flux measurements (*8*), and studies of volcanogenic massive sulfide (VMS) deposits, which represent ancient analogs for modern hydrothermal systems that have been tectonically uplifted and are now exposed on land (*9*). The numerical models provide the clearest depiction of a modern hydrothermal system but are theoretical and rely heavily on assumptions and generalizations regarding the structural and thermal properties of the underlying crust and magmatic heat source. Seafloor heat flux models are limited to providing two-dimensional hydrothermal fluid flow information along the surface of the seafloor, and the ancient VMS systems have been tectonically deformed and altered, making it difficult to reconstruct their original geometry.

**Fig. 1:**
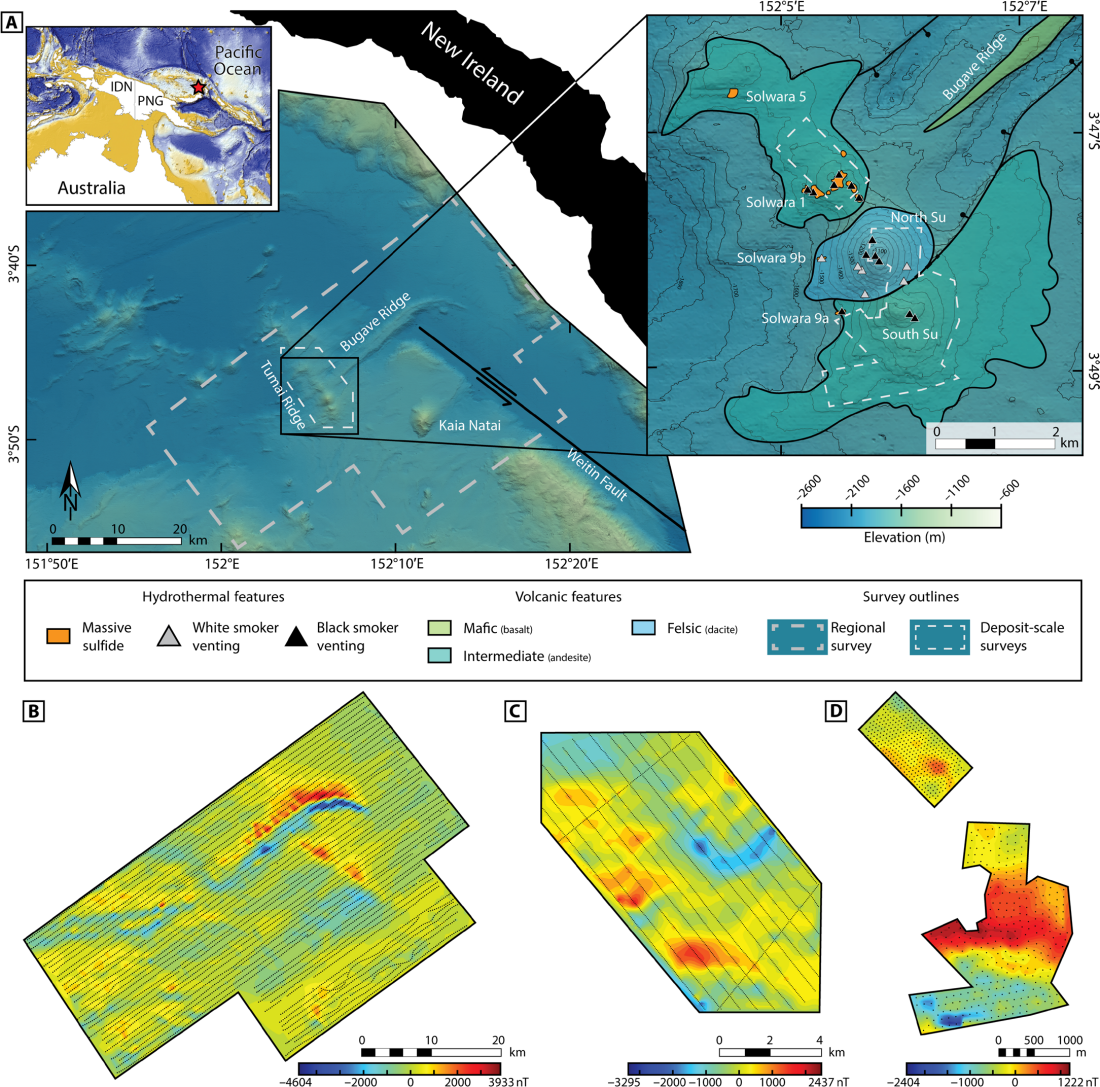
A map of the East Manus Basin in the region of the Tumai and Bugave Ridges, along with this study’s total magnetic field anomaly maps. (**A**) A 35-m resolution bathymetric map of the East Manus Basin, centered about the Tumai Ridge and Bugave Ridge intersection. The inset map shows the major geological features near Susu Knolls (*50*) and the locations of the known active and inactive hydrothermal vent sites along the Tumai Ridge (*22*, *30*), with 50-m bathymetric contour lines. Dashed gray and white lines represent the extents of the magnetic surveys. (**B** to **D**) Total magnetic field anomaly maps for the regional survey and both deposit-scale surveys, respectively. The black dots mark the positions of the measurement locations.

Changes to the magnetic properties of oceanic crust caused by alteration of primary minerals along high-temperature hydrothermal fluid circulation pathways can provide an alternative means to model subseafloor fluid flow geometries (*10*). In hydrothermal systems hosted within mafic to felsic volcanic rocks, the alteration of titanomagnetite to titanomaghemite, as well as the dissolution of titanomagnetite and subsequent formation of pyrite, by high-temperature fluids produces zones of anomalously low magnetic susceptibility and magnetization (*11*, *12*). Three-dimensional (3D) minimum-structure inverse modeling can be used to model the location and geometry of these anomalously low magnetism zones within the crust (*10*, *13*). Minimum-structure inversion of a magnetic field dataset constructs a 3D distribution of effective magnetic susceptibility in the seafloor that closely reproduces the measured survey data and that contains only sufficient features to reproduce those data (*14*). In this study, we applied minimum-structure inverse modeling to seafloor total magnetic field datasets centered on the Solwara 1 SMS deposit in the East Manus Basin, off the coast of Papua New Guinea.The East Manus Basin is an actively opening back-arc basin in the Bismarck Sea (Fig. 1A). Back-arc rifting is associated with the active New Britain Trench that borders the southern extent of the basin (*15*). To the north, the basin is bounded by the inactive Manus Trench. Within the basin, the crust is primarily composed of basaltic andesite, with intermittent concentrations of dacite, basalt, and andesite (*16*). Rifting is focused along the eastern Manus volcanic zone, which comprises a series of relatively short, oblique, spreading ridges separated by large transform offsets, resulting in considerable extensional faulting throughout the basin (*17*). Faulting is further enhanced by widespread volcanism associated with these tectonic features, resulting in a highly permeable linear fault and fracture network that is exploited by hydrothermal fluids. This results in the generation of numerous hydrothermal vent fields and SMS deposits along the length of the East Manus Basin, including PACMANUS (*18*) and DESMOS (*19*), and the Solwara 1 deposit (located on the Suzette volcanic edifice), located within Susu Knolls (*20*, *21*). Within the region of Susu Knolls, all the known SMS deposits are located along the Tumai Ridge, a volcanically active suture aligned orthogonal to the basin’s northwest by southeast extension. The concentration of SMS deposits along the ridge suggests that a shallow magmatic heat source below the ridge drives hydrothermal circulation (*22*).

The variation in crustal magnetic properties in the East Manus Basin was modeled through the inversion of total magnetic field data collected during an autonomous underwater vehicle (AUV) survey in 2006 (*23*) and two deep-tow surveys (2007 and 2016) that formed part of an SMS exploration program conducted by the Woods Hole Oceanographic Institute and Nautilus Minerals Inc. The deep-tow surveys covered the largest areas (5 km by 9 km and 44 km by 24 km at an average line spacing of 500 and 200 m, respectively, and at an altitude of 40 m; Fig. 1, B and C). The AUV data consist of two sets of grids focused directly over the Solwara 1 deposit and the North and South Su mounds (Susu Knolls; Fig. 1D) at an average line spacing of 50 m and an altitude of 20 m. Together, these three datasets allow the 3D magnetic structure of the crust to be modeled at both regional- and deposit-scale resolutions.

To numerically represent the seafloor in the East Manus Basin, a tetrahedral discretization of the subsurface was used (Fig. 5). Designing a model of the study site using tetrahedra, rather than the classically used rectangular cuboids (*10*), allowed the model to accurately fit the variable seafloor topography in this region. In addition, using a tetrahedral discretization allows regions of interest, such as below the measured data, to be modeled to a higher resolution than the rest of the model, optimizing computational efficiency and model accuracy. We inverted for scalar magnetic susceptibility, assuming that the total magnetization direction was parallel or antiparallel to the geomagnetic inducing field. The magnetic property modeled is then an “effective” susceptibility that combines both induced and remanent magnetization (*24*). This assumes that all remanent magnetization is parallel or antiparallel to the inducing field. On the basis of previous work in the East Manus Basin, this is considered a reasonable assumption within our study region (*25*), where there is minimal deformation of the seafloor crust that would change the orientation of any remanent magnetization with respect to the geomagnetic inducing field vector.

To date, magnetic modeling of the seafloor has been limited to the shallow subseafloor at hydrothermal discharge sites (*10*, *11*, *13*, *26*). Our study significantly improves upon the scale of these previous magnetic models by imaging the entire structure of a high-temperature convection column, including the identification of the top of the underlying magma chamber. The inversion modeling can identify such deep crustal features because of the large area that the regional deep-tow dataset covers (44 km by 24 km). Magnetic features located kilometers below the seafloor will impart very weak but very broad magnetic signatures due to the large distance between the feature and the measurement location. This contrasts with near-surface magnetic features that would produce very strong but narrow magnetic field signatures when measured at a similar distance above the seafloor. Therefore, having a dataset that samples the magnetic signatures of deep crustal features over a large area allows the broad, low-amplitude signals to be adequately measured and, therefore, modeled. This assumed that the magnetic features are large enough to produce a broad, low-amplitude signal that can be measured above noise levels.

## RESULTS

### Regional modeling

**Fig. 2:**
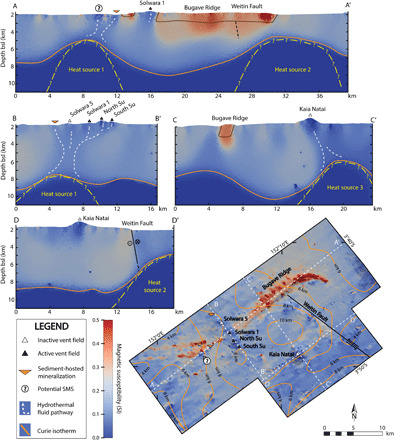
Map and cross sections of effective magnetic susceptibility from the regional 3D magnetic inversion model. A-A′ displays two prominent magnetic lows at ~2 km below the seafloor, which we interpreted to be magma chambers. The southwest magma chamber is the primary heat source driving hydrothermal circulation at Solwara 5 and Susu Knolls. B-B′ shows subseafloor high-temperature fluid pathways that feed the venting sites along the Tumai Ridge. C-C′ and D-D′ are two cross sections that pass through the hydrothermally active Kaia Natai volcano. The depth to the Curie isotherm is included on the plan view image as a contour map with orange lines, mapping the geometry of the underlying magmatic bodies.

Our most extensive model spans the length of the Tumai and Bugave Ridges, including the Kaia Natai volcano and the northern extent of the Weitin Fault (see Fig. 2 for the model and Fig. 1 for its bathymetry). The plan view of the 3D magnetic model highlights the regions of lowered effective magnetic susceptibility that correspond with Susu Knolls, Solwara 5, and Kaia Natai hydrothermal vent fields. In addition, the basaltic Bugave Ridge stands out as a magnetic high, because the relatively younger mafic to intermediate volcanics that makes up the ridge will contain more unoxidized titanomagnetite (*27*) than the surrounding older volcanic rock. In cross sections A and B of Fig. 2, we interpret the subseafloor zones of low effective magnetic susceptibility to represent pathways of hydrothermal fluid upflow below Solwara 5, Susu Knolls, and an unnamed site of sediment-hosted base and precious metal anomalies. These upflow zones appear to originate from a common heat source that is approximately 3 km to the west of Solwara 5, at a depth of 3 km below the seafloor (5 km below sea level; see Fig. 2).

To the west of Susu Knolls lies a region with 40 to 60 m of sediment cover known as West Su (*16*), with shallowly intruded mafic sills marked by areas of high effective magnetic susceptibility (seen near the two sites of sediment-hosted metal anomalies and the one possible SMS site labeled in Fig. 2). Sites of subsurface sulfide mineralization identified in this region through the gravity coring sediment collection method are underlain by a zone of low magnetization that are also interpreted to represent hydrothermal fluid pathways connected to the same heat source driving the Tumai Ridge’s hydrothermal activity (Fig. 2).

The heat sources identified from the regional model are labeled as heat sources 1 to 3. Heat source 1 lies approximately 3 km below the seafloor to the southwest of Susu Knolls and is connected to venting sites along the Tumai Ridge and the sediment-hosted sites to the west by magnetically imaged demagnetized alteration pipes (Fig. 2). Heat source 2 is the top of an interpreted magma chamber that spans the length of the Weitin Fault segment. Heat source 3 is approximately perpendicular to the Weitin Fault, lying 2 to 4 km below the seafloor to the south of Kaia Natai.

The Kaia Natai volcano, located southeast of Susu Knolls and independent of the Tumai and Bugave Ridges, hosts a low-temperature hydrothermal vent field (*28*). The presence of hydrothermal alteration at this site is supported by a low effective magnetic susceptibility region that extends approximately 2 km below the volcano (Fig. 2).

### Deposit-scale modeling

**Fig. 3:**
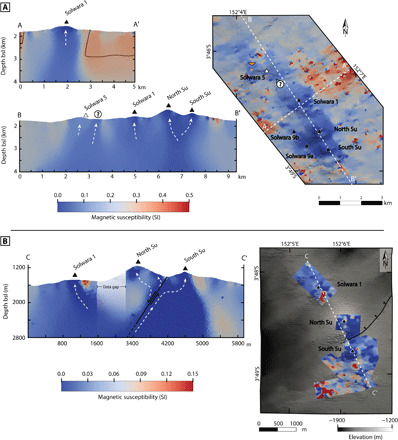
Map views and cross sections of the two deposit-scale 3D magnetic models. (**A**) The 2007 data inversion model, with cross sections: A-A′ shows a vertical alteration column associated with ascending hydrothermal beneath Solwara 1; B-B′ shows variation in near-surface effective magnetic susceptibility along the Tumai Ridge, with focused low susceptibility zones positioned at Solwara 5 and the three venting sites of Susu Knolls. An additional site of possible mineralization, southeast of Solwara 5, is interpreted from its magnetic characteristics, which match those of nearby known hydrothermal venting sites. (**B**) The 2006 AUV data inversion model, with a single cross section C-C′ showing partitioning of the fluid pathways within Susu Knolls leading to known vent sites. All symbols from the legend of Fig. 2. bsl, below sea level.

The two inversion models centered over the Solwara 1 deposit were derived from magnetic surveys collected over a smaller area but at greater resolution than the regional survey and therefore are able to better resolve near-surface magnetic features at the expense of deeper features. Where our regional model could define alteration zones associated with hydrothermal fluid channels from heat source to seafloor, the more detailed models better illustrate the individual fluid pathways near the seafloor after they diverged from a common upflow zone (Fig. 3). The models indicate a more intense magnetic signature below North and South Su, compared to Solwara 5, possibly indicating greater degrees of titanomagnetite destruction (Fig. 3A). At Solwara 1, the area of mineralization is resolved at even greater detail by inverting magnetic data collected near the seafloor by an AUV (Fig. 3B). Here, the pronounced magnetic low is due to the massive sulfide layer and underlying stockwork system that have lower effective magnetic susceptibilities than the surrounding altered host rock (*29*). Additional locations of further magnetic reduction are visible at sites of present (*30*) and past (*22*) hydrothermal venting, particularly around the top of North Su and the rim of South Su where known high-temperature venting occurs, as well as between the two volcanoes where lower temperature white smoker venting has been observed (Figs. 1 and 3B).

## DISCUSSION

High-temperature fluid pathways associated with subseafloor hydrothermal alteration, imaged using the magnetic inverse modeling, correlate with the known locations of both active and inactive hydrothermal vent sites or other areas with known surface mineralization associated with hydrothermal venting. Fluid pathways can be traced to heat sources 2 to 4 km below the seafloor. A further two sites of possible mineralization above altered upflow zones have been identified, illustrating the potential of magnetic inverse modeling as an effective exploration tool for both active and inactive sites of hydrothermal mineralization (Figs. 2 and 3A). These sites are in close proximity to other hydrothermal features yet have their own separate discordant zones of demagnetization. In the case of the interpreted SMS anomaly in Fig. 3A, a shallow 3D seismic survey collected by Nautilus Minerals identified that location as a site of possible mineralization, further supporting this interpretation.

Along the base of the regional model lies a surface of zero effective magnetic susceptibility, representing the Curie isotherm, beyond which the partially crystallized rock is too hot to maintain any magnetization (Fig. 2). The crust’s primary magnetic minerals, magnetite and titanomagnetite, have respective Curie temperatures of 530° to 585°C (*31*) and 110° to 170°C (*32*), respectively. The regional model indicates three zones with a prominent upwelling of the Curie isotherm, observed to the southwest of Susu Knolls (labeled heat source 1), along the Weitin Fault (heat source 2) and south of Kaia Natai (heat source 3; Fig. 2). These zones of upwelling are interpreted to indicate the presence of shallow magma chambers that warp the isotherms and create steep thermal gradients above the chambers (*6*, *33*–*35*). The melt underlying the Tumai Ridge has an estimated temperature of ~1010°C (*36*), which is much higher than the oceanic crust’s Curie point, but due to the high thermal gradient above magma chambers (*6*), the depth to the melt lens will likely be near to the Curie isotherm. Therefore, the peaks of the warped Curie isotherm are interpreted to represent the tops of the magma chambers.

Before this study, magmatic bodies had not been modeled through magnetic inversion because of the lack of seafloor magnetic surveying performed and modeled at this scale. Traditionally, the tops of magma chambers have been imaged through seismic and magnetotelluric methods and typically occur 2 to 3.5 km below the seafloor along spreading ridges (*37*). Specific to the Susu Knolls volcanic system, melt inclusion analyses have indicated the magma chamber to be 1 to 5 km below North Su (*36*), which, along with the typical depths of 2 to 3.5 km from other geophysical methods, is consistent with our interpretation.

The boundary between pillow basalts and underlying sheeted dykes [layer 2A/2B boundary (*38*)] in oceanic crust typically occurs 400 to 1000 m below the seafloor (*39*). Below the Tumai ridge, this depth range aligns with a branching of the near vertical hydrothermal alteration column, marking the zone where the fluid pathways separate toward the individual hydrothermal vent sites (Fig. 2 cross section B-B′). This suggests that rising hydrothermal fluids closely follow the higher-permeability regime aligned with semivertical dykes within the sheeted dyke zone. Once the rising fluids cross the layer 2A/2B boundary and enter the layer of crust dominated by more permeable sheet flows, hyaloclastite, and pillow basalts, the fluid flow gains a greater horizontal component and branching potential (*40*, *41*). In addition, normal faults in the shallow subseafloor on either side of the Bugave Ridge [see Figs. 1A (inset) and 3B] provide additional permeability.

**Fig. 4:**
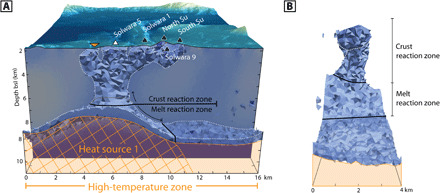
A 3D model of the high-temperature hydrothermal upflow column below the Tumai Ridge. The shown cross section is B-B′ from Fig. 2, with the alteration column visualized with a 0.12 SI threshold of the regional model’s effective magnetic susceptibility. (**A**) View of the column facing northeast. (**B**) View of the same column facing northwest. All surface hydrothermal feature symbols and the color scale follow the legend in Fig. 2.

Below the Tumai ridge and associated hydrothermal venting sites, the top of the zero effective magnetic susceptibility volume, representing the upper extent of the magma chamber, can be considered a lower boundary to the hydrothermal convection cells that feed the surface vent sites, as the melt will be an impermeable thermal boundary to hydrothermal fluids (*42*). Laterally, the width of the column of high-temperature fluids rising from the magma chambers can be approximated by the regions of anomalously low effective magnetic susceptibility defined by a model-derived upper threshold of 0.12 SI, in contrast to the background region’s susceptibility of approximately 0.2 SI (Fig. 4). Below this threshold, vertical connectivity along the high-temperature upflow column is no longer present. For an upper threshold, the convection column is no longer confidently distinguishable from the surrounding crust above a threshold of 0.14 SI. To study the minimum volume of crust that is exposed to high-temperature hydrothermal fluid convection, and because minimum-structure inverse modeling tends to produce blurred results, a lower approximate effective magnetic susceptibility value of 0.12 SI was chosen.

The resulting 3D model of the convection column beneath the Tumai Ridge demonstrates the connectivity and geometry of the fluid pathways with respect to its heat source and the overlying hydrothermal vent sites. Two prominent zones can be distinguished from this model: the first being the crust reaction zone (CRZ), where the high-temperature hydrothermal fluids are interacting with solely the oceanic crust, and the second being the melt reaction zone (MRZ), where, in addition to reacting with the crust, the hydrothermal fluids also circulate near the magma chamber and are exposed to magmatic fluids devolatilizing from the rapidly cooling and fracturing chamber margins, changing the chemistry of the fluids (*30*, *42*–*44*). This supercritical interaction with magmatic volatiles has been shown to enrich the fluids with precious (Au and Ag) and base metals (Cu, Zn, and Fe) (*42*), leading to greater likelihood of higher-grade SMS deposits forming at the overlying hydrothermal vent fields. Using the regional inversion model, the volumes of CRZ and MRZ can be measured and used to approximate the volume of rock that the high-temperature hydrothermal fluid interacts with. Under the Tumai Ridge, the CRZ was found to encompass approximately 4.7 ± 0.7 km^3^ and the MRZ 5.8 ± 0.6 km^3^, with the uncertainty being derived from a ±0.02 SI variation from the chosen 0.12 SI threshold.

In summary, regional, 3D inverse modeling of near-seafloor magnetic field data was used to image high-temperature hydrothermal fluid pathways from magmatic heat source and MRZ through the CRZ to seafloor fluid vent sites and associated SMS deposits. Knowing the 3D geometry of these systems increases our understanding of the scale and connectivity of hydrothermal upflow zones, allowing us to better identify the location of undiscovered vent fields, the volume of crust that the fluids interact with, and the spatial relationship between hydrothermal discharge sites that support unique biological communities. The limitation of this method is that it relies on imaging the alteration associated with high-temperature fluid-rock interactions and thus can only image high-temperature hydrothermal upflow and discharge at the seafloor but cannot image fluid pathways associated with colder hydrothermal recharge.

## MATERIALS AND METHODS

The inversions were performed with a minimum-structure modeling program developed at Memorial University (*45*). This program uses a tetrahedral discretization of the subsurface to recover a distribution of effective magnetic susceptibility that, when in the presence of the Earth’s field, generates an induced magnetic field that closely matches observed magnetic field measurements.

### Effective magnetic susceptibility

Effective magnetic susceptibility, χ_eff_, is the combination of scalar magnetic susceptibility, χ, and magnetization, **M**

$$\begin{array}{cc} B & =\chi B_{o}+\mu_{o}M\\ & =(\chi +\mu_{o}\frac{M\cdot B_{o}}{∥B_{o}∥})B_{o}\\ & =\chi_{\text{eff}}B_{o} \end{array}$$ (1)

which can be accurately combined when the natural remanent magnetization, **M**, in the surveyed region is parallel or antiparallel to the Earth’s inducing field, **B**_**o**_. In the International System of Units (SI), the magnetic field unit is tesla, T, the magnetization unit is amperes per meter, A/m, and the permeability of free space, μ_o_, is equal to 4π × 10^−7^ Tm^2^/A.

### Objective function and regularization

The inverse problem is underdetermined, resulting from there generally being many more cells in the discretized mesh than there are observed data points. The problem is also ill-posed, which results from several factors, including the presence of noise in the data and the natural decay in model sensitivity with distance from the observation points. Hence, it is not enough to only fit the data, and measures of model structure must be put in place to provide unique and reliable solutions to the inverse problem. An objective function is minimized to solve for a model that not only fits the data but also is geologically realistic

$$\Phi =\Phi_{d}+\Phi_{m}$$ (2)

with data misfit

$$\Phi_{d}=\frac{1}{N}\Sigma_{i=1}^{N}\frac{{\left(d_{i,\text{pred}}-d_{i,\text{obs}}\right)}^{2}}{\sigma_{i}^{2}}$$ (3)

and model measure

$$\Phi_{m}=v_{c}^{T}W_{c}\rho (\omega )$$ (4)

in its generalized form. The data misfit term is normalized by the number of data measurement points, *N*, and is composed of the difference in the magnetic field signal from the inversion model, *d*_*i*, pred_, and the measured magnetic field at that same point, *d*_*i*, obs_, with signal noise σ*_i_*. The general model measure term is composed of a product of the vector of the inverse model’s cell volumes, $v_{c}^{T}$, the weighting matrix, ***W****_c_*, and a vector holding the physical property information, ρ(**ω**). The main purpose of the model measure term is to minimize the amount of unnecessary structure generated during the inversion modeling. Following Occam’s Razor, the simplest model that provides an adequate fit to the observed data is most likely to be the most geologically accurate (*14*).

To preferentially weight cells in the model with low sensitivities, a sensitivity weighting (*46*) was used in our model misfit whose matrix was composed of elements

wj=(Σi=1N∣Gijvj∣2)12 (5)

for the *j*th model cell that has a volume of *v_j_* and a sensitivity of *G_ij_*, with respect to the *i*th observation point. ***G*** is the sensitivity matrix for the inverse problem. This sensitivity weighting helped to prevent the inverse problem from creating a trivial solution of purely near-surface magnetic anomalies.

In place of the classically used L2 norm (*14*, *47*)

$$\rho (D_{f}m)={\left(D_{f}m\right)}^{2}$$ (6)

used to smooth the effective magnetic susceptibility distribution by applying a general difference matrix, ***D***_***f***_, to the model vector, ***m***, and limit the nonuniqueness inherent to inversion modeling, we used a total variation-like measure

ρ(ωt)=(ωt2+ϵ2)p2 (7)

with

$$\omega_{t}=Q_{x}({\left(D_{x}m\right)}^{2})+Q_{y}({\left(D_{y}m\right)}^{2})+Q_{z}({\left(D_{z}m\right)}^{2})$$ (8)

to promote more discrete features within the magnetic susceptibility models. The total variation measure contains three gradient operators, ***D****_k_* (*k* = *x*, *y*, *z*), for each cartesian direction, and three matrices, ***Q****_k_* (*k* = *x*, *y*, *z*), which interpolate the *k*-direction squared gradient values at the model cell centers. During all our inversions, the parameter values used were ϵ = 10^−9^ and *p* = 1.05.

To initiate the inversion, we used a homogeneous 0.2 SI effective magnetic susceptibility starting model. No reference models were used during the inversion, as the subseafloor geology in the East Manus Basin is fairly unknown, so we did not want to include any potentially incorrect bias into the modeling. To constrain the inversion, positivity was enforced with respect to the effective magnetic susceptibility. This was done to generate more geologically representative models, as models that we created without positivity were almost entirely positive, save for very small-scale model features indicative of data overfitting. It is possible to have negative effective susceptibility, which would be the case where the remanent magnetization component of the effective susceptibility outweighs the induced field component while being antiparallel to the Earth’s present field. If this were the case in this study’s models, then there would be larger regions of negative effective susceptibility, spanning zones of homogeneous rock and/or alteration types, which were not present.

### Mesh design

**Fig. 5:**
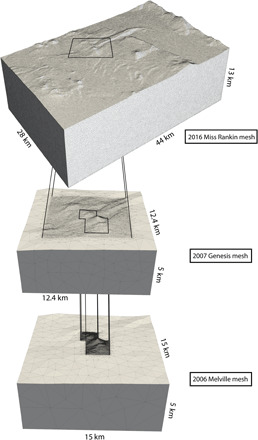
The three meshes used for our inverse modeling, shown in relation to each other. At the top is the mesh for the regional inversion model, from the 2016 *M/V Miss Rankin* cruise, with the two deposit-scale meshes below it in descending order of survey size, from the 2007 *M/V Genesis* then the 2006 *R/V Melville* cruises. All meshes are viewed from the south.

Each inversion was performed on a separate model mesh, whose average cell size in the volumes of interest was dependent on the density of the respective dataset. We first ensured that the density of triangular facets in the bathymetry surface, as generated with the program Triangle (*48*), was greater or equal to the density of observation points. Once a sufficiently dense surface mesh was made to fit the bathymetry of the region, the program TetGen (*49*) was implemented to discretize the full extent of the volume of interest with tetrahedra. The volume of interest for each model was designed such that its boundary outlined the extent of each survey’s data points, plus at least a 2-km-thick padded cell region surrounding the volume of interest (Fig. 5). These padded cells limit the edge effects resulting from approximating the seafloor as a smaller, discretely bounded block.

**Fig. 6:**
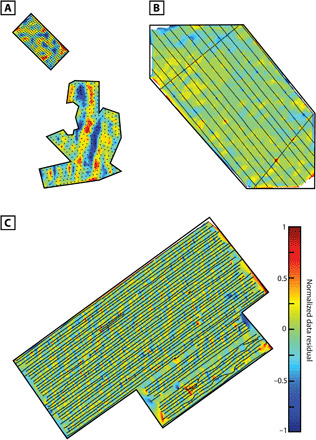
The normalized data residuals for the three inverted datasets. (**A**) Normalized data residual for the inverted 2006 AUV magnetic dataset, (**B**) for the inverted 2007 deep-tow magnetic dataset, and (**C**) for the inverted 2016 deep-tow magnetic dataset.

The Miss Rankin inversion was performed on a mesh of 1,792,073 cells and 7927 data observation points. The survey line spacing from the *M/V Miss Rankin* cruise was approximately 500 m, and the data were decimated to have a 200-m spacing along survey lines. The Genesis mesh contained 1,367,241 cells. We used 2368 data points, with an approximate line spacing of 400 m and an along-line spacing of 100 m. Last, the Melville inversion used a mesh of 1,345,270 cells and 717 data points. The AUV data over Susu Knolls were collected over four dives, with one dive providing a 14-line grid over the Solwara 1 deposit and 6 variably oriented and spaced grids over North Su and South Su. For the inversion, the data over the Solwara 1 deposit were decimated to 50-m along-line spacing, approximately equal to the average line spacing, and the North Su and South Su survey was decimated to 100-m point spacing (along-line and line spacing). See Fig. 1 for the positioning of each survey’s magnetic field measurements, with the normalized residuals displayed in Fig. 6.

### Model analysis

Analyzing the normalized data residual plots of Fig. 6, two prominent features are present at the peaks of the North and South Su mounds (Fig. 6A). These most likely result from an error introduced by the resolution of the bathymetry used to construct the mesh for that inversion. The available 35-m resolution led to the linear interpolation of features between those bathymetric points, and at locations like the peaks of the volcanoes linearly interpolating between points spaced 34 m apart can lead to noticeable difference from the true seafloor bathymetry. To generate the normalized data residuals, a data uncertainty of 1% was used, the same used during the inverse modeling. The amplitude of noise in the three magnetic datasets was unknown, so a percent noise of 1% was assumed for the inverted data, and then a normalized target data misfit of 64 was used to generate inversion models that did not produce artifacts indicative of data overfitting. This target misfit indicates that the noise, if assumed to be Gaussian, is approximately 8%.

The three magnetic surveys took place over a span of 10 years, so three different inducing magnetic field vectors had to be used during the inversions. At the time of the 2006 *R/V Melville* cruise at Solwara 1, the Earth’s magnetic field had a strength, inclination, and declination of (39,083 nT, *I* = −21.714°, *D* = 6.535°). The 2007 *Genesis* magnetic anomaly was calculated with a magnetic field vector of (39,102 nT, *I* = −21.683°, and *D* = 6.61°), and the 2016 *Miss Rankin* vector was (39,037 nT, *I* = −21.816°, and *D* = 6.201°). All field vectors were calculated using the Enhanced Magnetic Model (EMM2017).

All inversions were performed on a 24-core 2.20-GHz Intel Xeon E5-2650 processor, yielding run times of 325.1, 36.9, and 15.3 hours for the Miss Rankin, Genesis, and Melville models, respectively.
